# Research progress and strategies for vaccines and targeted drugs against highly virulent porcine epidemic diarrhea virus

**DOI:** 10.3389/fmicb.2025.1666167

**Published:** 2025-10-27

**Authors:** Qiuxuan Wang, Songrui Liu, Hongrui Chen, Xuanyi Liu, Hanjia Zhang, Xuehui Wang, Qingfeng Meng, Hao Dong

**Affiliations:** ^1^College of Life Sciences, Jilin Agricultural University, Changchun, China; ^2^Engineering Research Center of Bioreactor and Pharmaceutical Development, Jilin Agricultural University, Changchun, China

**Keywords:** porcine epidemic diarrhea virus, anti-virus, vaccine, drug, toll-like receptors 3

## Abstract

As an acute and highly contagious enteric disease of swine, porcine epidemic diarrhea virus (PEDV) has caused high piglet mortality and significant economic losses. Commercialized vaccines provide only partial cross-protection against the novel, highly virulent PEDV strains. Developing new vaccines against highly virulent PEDV strains would help protect the pig industry from the serious challenges posed by novel, highly virulent PEDV infections. Natural compounds and chemical and biochemical source-targeted drugs designed to act on specific proteins, enzymes, or mechanisms can complement each other’s advantages when used in combination, thereby enhancing the effectiveness of drug-based prevention in the control of highly virulent PEDV. Drugs targeting Toll-like receptor 3 (TLR3) can aid vaccines to compensate for interferon (IFN) secretory deficiencies to protect pigs from highly virulent PEDV infection. This review summarizes recent progress in the development of vaccines against highly virulent PEDV, natural compounds, and chemical and biochemical source-targeted drugs that have been explored in cell and pig models with clearly defined mechanisms. It also aims to provide comprehensive strategies for the prevention and control of highly virulent PEDV infections in pigs.

## 1 Introduction

As an acute and highly contagious enteric disease of pigs, porcine epidemic diarrhea (PED) can result in dehydration, vomiting, diarrhea, and severe enteritis. Its lethality is particularly high in suckling pigs (Stadler et al., 2015). Porcine epidemic diarrhea virus (PEDV), a causative agent belonging to the genus *Alphacoronavirus* in the family Coronaviridae, is an enveloped, positive, single-stranded RNA virus (Karte et al., 2020). The PEDV genome is approximately 28 kb and contains at least seven open reading frames (ORFs), which encode two large polyprotein precursors (pp1a and pp1ab); the spike (S), membrane (M), and envelope (E) structural proteins; and nucleocapsid (N) proteins, as well as an accessory protein, ORF3 (Zhuang et al., 2025). The virus genotype includes G1 (classical G1a and recombinant G1b) and G2 (local epidemic G2a and global epidemic G2b) (Jang et al., 2023).

The highly pathogenic (HP)-G2b PEDV caused a pandemic that severely impacted pig-producing nations in America and Asia during 2013–2014 and also threatened the global pig industry (Park et al., 2007; Puranaveja et al., 2009; Lee et al., 2024). Most PEDV strains isolated from Vietnam belonged to the genotypes G1 and G2 and had very high genetic similarity with strains isolated from China and Thailand (Nguyen et al., 2023). The virus was first recognized in Europe in the 1970s and caused high piglet mortality and significant economic losses in Germany, France, Belgium, Ukraine, Austria, Portugal, and the Netherlands in 2014 (Dastjerdi et al., 2015; Grasland et al., 2015; Mesquita et al., 2015; Stadler et al., 2015; Steinrigl et al., 2015; Theuns et al., 2015; Dortmans et al., 2018). It was reported that the prevalence of PEDV-positive piglets during the first week on Spanish farms ranged from 3.7 to 12.9% in 2014 (Mesonero-Escuredo et al., 2018; Vidal et al., 2019). A recent investigation of 106 Spanish pig farms between 2017 and 2019 showed that the detected PEDV rate was 38.7% (Monteagudo et al., 2022). The investigation showed that PED can rapidly spread in PEDV-negative herds and cause 100% morbidity and 30 to 90% mortality in piglets (Jang et al., 2023). PEDV can also cause a 12.6% reduction in the farrowing rate and result in a 5.7% failure-to-breed rate, a 1.3% abortion rate, and 2.0% mummified fetuses, negatively affecting the reproductive performance of mature sows (Weng et al., 2016).

To fight the novel highly virulent PEDV infection, this review summarizes recent progress in the development of vaccines against highly virulent PEDV, natural compounds, and chemical and biochemical source-targeted drugs that have been explored in cell and pig models with clearly defined mechanisms. It also aims to provide comprehensive strategies for the prevention and control of highly virulent PEDV infections in pigs.

## 2 Progress and strategies in vaccines against highly virulent PEDV

Viral entry, attachment, induction of neutralizing antibodies, and membrane fusion are mediated by the S1and S2 domains of the PEDV S glycoprotein. The CO-26 K-equivalent (COE) and N-terminal domain (NTD) in the S1 region are crucial neutralizing epitopes and potential co-receptor binding sites for the vaccine development of PEDV (Kirchdoerfer et al., 2021). Lipid nanoparticle (LNP)-encapsulated mRNA (mRNA-LNP) vaccines encoding a PEDV multiepitope chimeric spike (Sm) protein (PEDV-S mRNA-LNP) have been demonstrated to activate CD4 + and CD8 + T cells and induce PEDV-specific IgG and IgA in the serum and colostrum of S-mRNA-immunized sows, which could be transferred to suckling neonatal piglets, providing protection against AH2012/12 infection (Kirchdoerfer et al., 2021; Zhao et al., 2024).

Whole-virus vaccines in traditional PEDV vaccines include inactivated and attenuated vaccines. In contrast to traditional PEDV vaccines, subunit vaccines can provide safety, without viral nucleic acids, the redesigned antigens and multiple antigens combination with the adjuvant addition in immunity efficacy elevation (Du et al., 2016). A complete subunit vaccine production system would greatly facilitate a quick response to emergency epidemics (Li Z. et al., 2020). The study showed that the full-length S protein subunit vaccine could effectively induce high levels of S-specific IgG, IgA, and neutralizing antibodies in pigs infected with AH2012/12. It also increased the proliferation of peripheral blood mononuclear cells and increased interferon-*γ* (IFN-γ) and interleukin-4 (IL-4) expression levels in peripheral blood to reduce diarrheal index scores, fecal viral loads, and intestinal pathological damage in immunized piglets (Guo et al., 2024).

The addition of trypsin is crucial but also increases the complexity of vaccine production and cost in the propagation of PEDV. It has been reported that PEDV trypsin independence is associated with the S2′ site and Y976/977 of the PEDV spike (S) protein (Li M. et al., 2023). Li M et al. used AJ1102 and the trypsin-independent genotype 1 (G1) PEDV strain JS2008 to generate a recombinant PEDV carrying a chimeric S protein and successfully constructed the trypsin-independent PEDV strain rAJ1102-S2′JS2008 (Li M. et al., 2023). It was able to effectively replicate in the absence of trypsin and could induce neutralizing antibodies against AJ1102 and JS2008, providing protection to pigs against G1 and G2 PEDV infections (Niu and Wang, 2022; Li M. et al., 2023).

Immunizing sows with PEDV vaccines between 20 and 30 days will provide substantial passive immunity to their newborn piglets, especially mucosal immunity, which is essential for the sows (Lin et al., 2016). As a particle-mediated delivery system for vaccines, biodegradable and biocompatible poly (D, L-lactide-co-glycolic acid) (PLGA) nanoparticles (NPs) can protect the entrapped vaccine from protease-mediated degradation at mucosal surfaces and stimulate the underlying mucosal immune cells to provide protection for sows infected with AH2012/12 (Binjawadagi et al., 2014a; Binjawadagi et al., 2014b). PLGA nanoparticle-entrapped PEDV killed vaccine antigens (KAg) (PLGA-KAg) have been shown to improve PEDV-specific IgG and IgA antibody titers, induce lymphocyte proliferation responses, and increase IFN-γ levels in pregnant sows and their suckling piglets (Li et al., 2017).

As a potential adjuvant, flagellin can induce Th1 and Th2 mixed-cell responses (Li et al., 2018). Flagellin can be used in combination with inactivated or killed PEDV vaccines to elevate mucosal and systemic IgG and IgA levels, thereby protecting piglets from PEDV AH2012/12 infection (Xu X. et al., 2020).

For the highly virulent PEDV G2 strains, traditional vaccines can only provide partial cross-protection (Wang et al., 2016). Commercialized vaccines, including recombinant PEDV S protein, an inactivated whole-virus vaccine based on a non-S INDEL PEDV strain, and a subunit vaccine using HEK-293 T cell-expressed PEDV S1 proteins, have been used to control virulent G2 viruses in the United States (Makadiya et al., 2016). However, commercialized vaccines cannot provide consistency in stimulating solid lactogenic immunity to protect suckling piglets from G2 virus infection (Crawford et al., 2016). Virus-like particles (VLPs) can improve immunogenicity, drain freely into lymph nodes, and be efficiently taken up by antigen-presenting cells to promote CD4 + T helper cell and CD8 + cytotoxic T cell responses (Dudziak et al., 2007; Mohsen et al., 2017). As characterized nanoparticles of conformational epitopes, VLPs can induce the subsequent humoral immunity by interacting with B cells (Manolova et al., 2008; Hsueh et al., 2020). In the development of safe, effective, and economical vaccines against enteric viral diseases, VLP vaccines represent an important strategy by stimulating cellular, mucosal, and humoral immunity. In the current study, PEDV VLPs of CCL25/28 were demonstrated to protect pigs from PEDVPT-P7infection by increasing systemic anti-PEDV S-specific IgG, mucosal IgA, and cellular immunity (Leidenberger et al., 2017; Hsu et al., 2020; Lu et al., 2020).

mRNA-LNP vaccines, the full-length S protein subunit, the trypsin-independent genotype 1 (G1) PEDV JS2008 strain, PLGA nanoparticle-entrapped PEDV killed vaccines, flagellin, and PEDV VLPs of CCL25/28 have shown different immune regulation efficiencies in enhancing systemic anti-PEDV S-specific IgG, mucosal IgA, and cell immunity to protect pigs from highly virulent PEDV infection (Table 1). To continue exploring vaccines, it is indispensable to prevent and control PED infections caused by different novel highly virulent PEDV strains in pigs.

**Table 1:** Vaccines against highly virulent PEDV.

Vaccine	Immune induction	Protection efficiency	References
PEDV-S mRNA-LNP vaccine	PEDV-specific humoral and cellular immune responses.	Protected immunized piglets against the PEDV AH2012/12 strain	Kirchdoerfer et al. (2021) and Zhao et al. (2024)
Trimeric full-length S protein subunit vaccine	High levels of S-specific IgG, IgA, and neutralizing antibodies; increased expression levels of IFN-γ and IL-4.	Reduced intestinal pathological damage in immunized piglets infected with AH2012/12	Guo et al. (2024)
Recombinant rAJ1102-S2′JS2008 Vaccine	Induced neutralizing antibodies	Protected pigs from G1 and G2 PEDV infections.	Niu and Wang (2022) and Li M. et al. (2023)
PLGA-KAg	Improved lymphocyte proliferation responses, IFN-γ levels, and PEDV-specific IgG and IgA antibody titers	Provided protective immunity against PEDV AH2012/12 strain infection in suckling piglets.	Li et al. (2017)
A flagellin -adjuvanted inactivated porcine epidemic diarrhea virus (PEDV) vaccine	Elicited high levels of IgG, IgA, and neutralizing antibodies	Conferred protective immunity to piglets against PEDV strain AH2012/12 infection.	Xu X. et al. (2020)
PEDV VLPs with CCL25/28	Modulated the immune responses by enhancing systemic anti-PEDV S-specific IgG, mucosal IgA, and cell immunity	Alleviated clinical signs in piglets infected with PEDVPT-P7.	Hsu et al. (2020) and Lu et al. (2020)

## 3 Progress and strategies in drugs targeting highly virulent PEDV

Maternal antibodies from colostrum and milk are important to protect piglets from PEDV infection (Leidenberger et al., 2017). PEDV mutation could decrease the full protection of the vaccines (Li S. et al., 2020). Therefore, it is necessary to update vaccines based on prevalent PEDV strains and explore new strategies (Yang et al., 2023). Antiviral natural compounds from plant extracts and Chinese herbal medicines have been increasingly demonstrated in recent years. In view of rich sources, unique chemical structures, and diverse activities of natural compounds in the development of new anti-highly virulent PEDV drugs, natural compounds will compensate for the vaccine deficiency in against PEDV prevalent strains (Russo et al., 2020; Gong et al., 2023). Recently, the anti-highly virulent PEDV of natural products target drugs have become a hot spot because of its lower side effects, cheaper investment and avoidable risk in developing resistance (Behzadi et al., 2023; Liang et al., 2024). Many natural compounds have also been reported to be effective in inhibiting highly virulent PEDV (Sun et al., 2022). Drugs including flavonol, tomatidine, and wogonin have been reported to affect highly virulent PEDV by interacting with the Mpro or 3CLpro proteins of PEDV *in vitro* (Wang et al., 2020; Wang J. et al., 2023; Liang et al., 2024) (Table 2). These compounds can be good candidate drugs against highly virulent PEDV in cells or pigs, pending further demonstration in *in vivo* studies. Based on their effects on highly virulent PEDV *in vitro and vivo*, licorice extract, buddlejasaponin IVb, hypericum japonicum extract, puerarin, and aloe extract have been shown to inhibit highly virulent PEDV by interfering with the N protein, ORF3 mRNA, and M protein; inhibiting the PI3K and NF-κB signaling pathways; and blocking the transcription of viral N genes (Wu et al., 2020; Xu Z. et al., 2020; Su et al., 2021; Sun et al., 2022; Rao et al., 2023; Yang et al., 2023). These drugs could reduce the replication of highly virulent PEDV and also alleviate clinical symptoms in pigs. They hold promising clinical value for future exploration of their effects against highly virulent PEDV both *in vitro* and *in vivo*.

**Table 2:** Natural compounds targeting highly virulent PEDV.

Natural compounds	Inhibit PEDV in cells	Effective in pigs	Target	References
Flavonol	PEDV-YN145	No report	Interacts with PEDV M^pro^.	Liang et al. (2024)
Tomatidine	PEDV MS, YZ, SH, and CV777	No report	Inhibition of 3CLpro activity.	Wang et al. (2020)
Wogonin	PEDV AH2012/12	No report	Exerts an inhibitory effect on M^pro^.	Wang J. et al. (2023)
Licorice extract	PEDV HM2017	Against PEDV HM2017 infection in piglets.	1. Interfering with the PEDV N protein, ORF3 mRNA, and M protein 2. Inhibiting the PI3K signaling pathway	Yang et al. (2023)
Buddlejasaponin IVb	PEDV AH-2018-HF1	Relieving clinical symptoms in pigs.	Inhibits the NF-κB signaling pathway.	Sun et al. (2022)
Hypericum japonicum extract	PEDV-CV777 and PEDV-G2	Improving intestinal pathology in piglets	Interfering with interaction between the N protein and p53.	Rao et al. (2023)
Puerarin	PEDV Yunnan province strain	Reduction of PEDV impact on growth performance in piglets.	Regulating the IFN and NF-κB signaling pathways.	Wu et al. (2020)
Aloe extract	PEDV GDS01	Protects newborn piglets from PEDV variant GDS01 infection	Blocking the transcription of viral N genes.	Xu Z. et al. (2020)

There are chemical drugs targeting highly virulent PEDV, including niclosamide, tubercidin, and ivermectin, that can inhibit the proliferation of highly virulent PEDV *in vitro* by targeting the specific viral mechanisms (Wang Y. et al., 2023; Wang et al., 2024; Xu et al., 2024) (Table 3). Considering the evasive strategies of PEDV, it is important to regulate the proliferation of highly virulent PEDV by targeting the signal transducer and activator of transcription 3 (STAT3) and RNA-dependent RNA polymerase (RdRp) (Wang Y. et al., 2023; Wang et al., 2024; Xu et al., 2024). Although these targeted drugs have only been tested *in vitro*, they still offer extraordinary therapy strategies for the prevention of highly virulent PEDV. Among these drugs, PA-824 has been demonstrated to inhibit the proliferation of highly virulent PEDV and alleviate diarrhea symptoms in pigs caused by PEDV AH-2018-HF infection by suppressing PEDV-induced p53 activation *in vitro* and *in vivo* (Li et al., 2024). Especially, the specially target drugs tested in pigs will be priority in synergistic therapy and increase anti-highly virulent PEDV efficiency.

**Table 3:** Chemical drugs targeting highly virulent PEDV.

Drug	Inhibit PEDV in cells	Effective in pigs	Target	References
Niclosamide	CV777, HNXX, HB, HW, and recombinant PEDV strains DR13-GFP and DR13-Rlu	No report	Targeting STAT3.	Wang Y. et al. (2023)
Tubercidin	CV777, HNAY, HNXX, HB, DR13-GFP, and GDU-GFP	No report	Forms hydrophobic interactions with the RdRp of PEDV.	Wang et al. (2024)
Ivermectin	CV777, HW, HNXX, HB (GII subtype), and PEDV (DR13-GFP)	No report	Induces cell cycle arrest to inhibit viral release.	Xu et al. (2024)
PA-824	PEDV AH-2018-HF	Reducing the pathogenic effect of PEDV in piglets	Suppressing PEDV-induced p53 activation.	Li et al. (2024)

Biochemical source drugs, including RNA G-quadruplexes and Karyopherin *α*2 (KPNA2), have shown substantial inhibition of highly virulent PEDV replication by targeting the G4 structure in Nsp5 and the E protein, respectively, *in vitro* (Gao et al., 2023; Li Y. et al., 2023). The highly virulent PEDV genome and structural proteins (S, E, M, and N) (Table 4) are crucial determinants of the molecular epidemiological characteristics of PEDV (Karte et al., 2020; Jang et al., 2023; Zhuang et al., 2025). It is necessary to further explore and test biochemical source drugs in vivo, as they may provide new options to face emerging challenges from PEDV variant strains. As an important direction for future studies, there is a real demand in veterinary clinics to explore and screen high-efficiency, low-toxicity, and low-residue drugs with targeted therapy against highly virulent PEDV (Behzadi et al., 2023). Natural compounds and chemical and biochemical source-targeted drugs can complement each other’s advantages through drug combination, thereby promoting the efficacy of drug-based prevention and control of highly virulent PEDV.

**Table 4:** Biochemical source drugs targeting highly virulent PEDV.

Drug	Inhibit PEDV in cells	Effective in pigs	Target	References
RNA G-quadruplexes	CV777, HNAY, HW, and DR13-GFP strains	No report	Targeting the G4 structure in Nsp5.	Li Y. et al. (2023)
KPNA2	PEDV strain GDS01	No report	Degrading the viral E protein by autophagy	Gao et al. (2023)

## 4 Progress and strategies in drugs targeting toll-like receptor 3 (TLR3)

**Figure 1:**
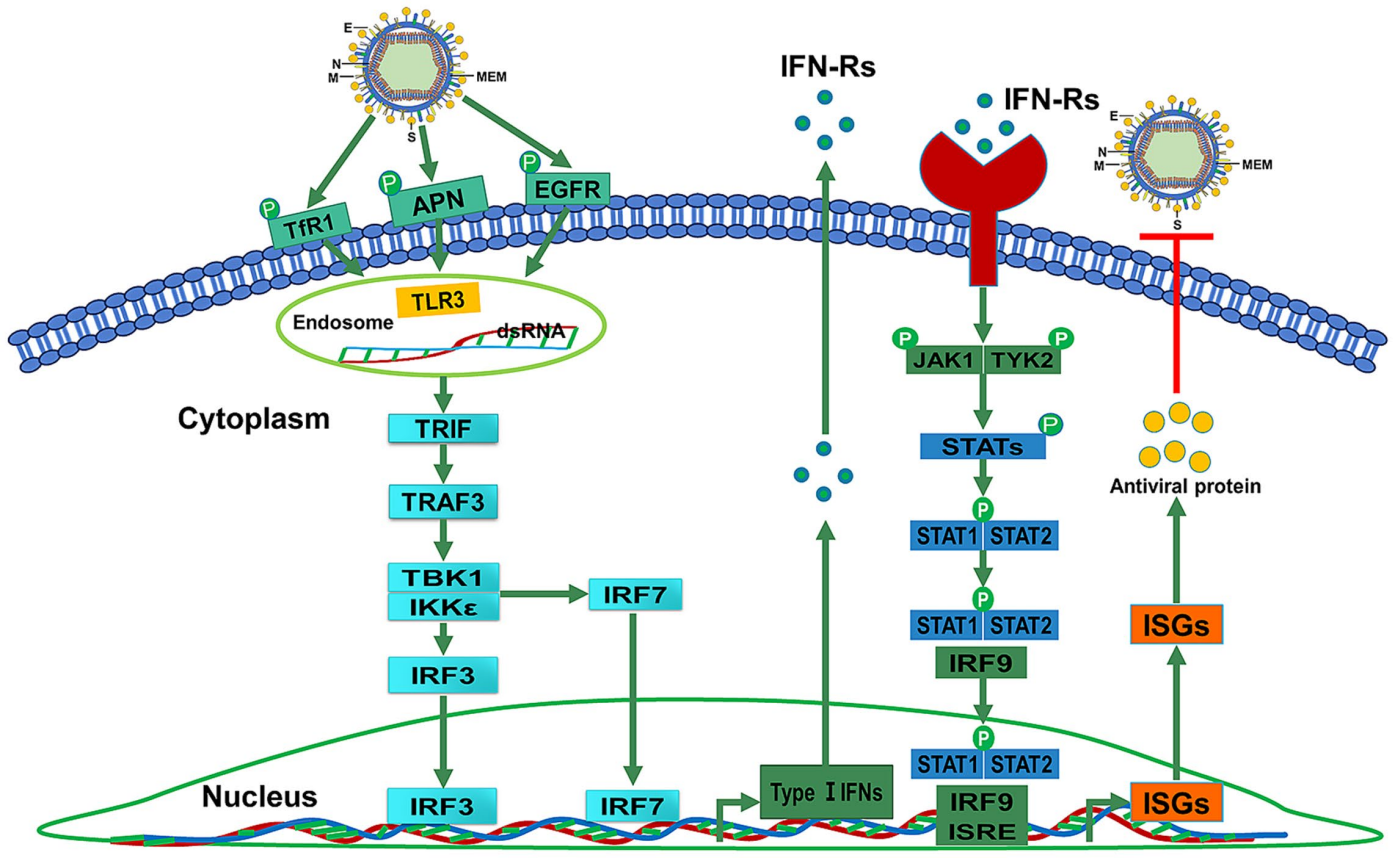
TLR3 upregulates IFN-*α*/*β* expression and induces AVP synthesis activity by recognizing dsRNA. The TLR3-mediated signaling pathway includes the TRIF-dependent NF-κB and IRF3/7 pathways. TRIF interacts with TRAF3 to elicit a cascade of reactions to induce the production of IFNs-1.

Inducing antiviral innate immune and inflammatory responses is an important precondition for repelling viral infections (Yang and Shu, 2020). Studies have shown that the production of type I or type III IFNs could be inhibited by highly virulent PEDV N proteins, such as nsp1, PLP2, nsp5, nsp15, and nsp16 (Deng et al., 2019; Shi et al., 2019). This inhibition benefits highly virulent PEDV by enabling immune evasion through suppression of IFN production pathways and disruption of transcription factor activation involved in IFN induction (Cao et al., 2015a; Guo et al., 2016; Li S. et al., 2020). Pattern recognition receptors (PRRs) can specifically recognize pathogen-associated molecular patterns (PAMPs) by activating IFN- and interleukin-1 (IL-1)-mediated proinflammatory responses in animals (Rai, 2020). As a member of the virus-perceiving PRRs, Toll-like receptor 3 (TLR3) can initiate downstream signal transduction, upregulate the IFN-*α*/*β* expression, and induce antiviral protein (AVP) synthesis activity by recognizing viral double-stranded RNA (dsRNA) (Unterholzner et al., 2010; Matsumoto et al., 2011). Within the TLR family, TLR3 is the only receptor that induces IFN-β production through the Toll/IL-1 receptor (TIR) domain-containing adapter-inducing interferon-β (TRIF) pathway (Yang and Shu, 2020). The TRIF-dependent nuclear factor-κB (NF-κB) and IFN regulatory factor 3/7 (IRF3/7) pathways are regulated by TLR3 (Matsumoto et al., 2011). When TLR3 is activated by viral dsRNA, TRIF could elicit a cascade of reactions by triggering tumor necrosis factor (TNF) receptor-associated factor 3 (TRAF3) and TRAF6 (Fang et al., 2013; Bugge et al., 2017) (Figure 1).

TLR3 localizes to endosomes and the cell surface in macrophages and mast cells (MCs) but is restricted to endosomes in myeloid dendritic cells (DCs) (Matsumoto et al., 2003; Matsumoto et al., 2011; Agier et al., 2016). Type I IFNs (*α* and β) are associated with viral clearance and can be produced by DCs (Matsumoto et al., 2003). TLR3 can drive antigen-presenting DCs to induce IFN production (Soto et al., 2020). TLR3 also promotes IRF3, type I and II IFN receptor, and major histocompatibility complex (MHC) I expression in MCs to enhance the cellular antiviral response (Soto et al., 2020; Witczak et al., 2020). Viral infection leads to TLR3 expression upregulation in DCs of mice and humans. Virus dsRNA is recognized by the TLR3 ectodomain (ECD) (Negishi et al., 2008; Abe et al., 2012). The TIR domain of TLR3 can recruit TRIF (Chattopadhyay and Sen, 2014). It can stimulate the phosphorylation of IRF3, which leads to the production of type I IFNs (Takeda and Akira, 2004). A study demonstrated that TLR3 positively contributes to NF-kB activation in response to PEDV infection (Cao et al., 2015b). TLR3 activates NF-κB signaling through TRIF-dependent conscription of two cascades. It is most noteworthy pathway of TLR3 signaling that can provoke TNF, IL-1, CCL2, CXCL8, endothelial adhesion molecules, and type I IFNs to against viruses (Komal et al., 2021). The TLR3 agonist can upregulate the expression of IFN-α/*β* and induce AVP synthesis activity by recognizing virus dsRNA to activate TLR3 downstream signal transduction (Unterholzner et al., 2010; Matsumoto et al., 2011).

Novel TLR3 agonists include RGC100, Poly-IC, and ARNAX. As a novel TLR3 agonist, RGC100 can target endosomal TLR3 and activate murine myeloid DCs to promote proinflammatory cytokine secretion in a dose-dependent manner (Naumann et al., 2013). Considering its immunological properties, RGC100 may represent a promising candidate for prevention and therapy vaccination strategies against PEDV. As a synthetic dsRNA analog, polycytidylic acid (PolyI:C) can be recognized by RIG-I/MDA-5 and TLR3 receptors to activate transcription factors that are responsible for the expression of type I IFNs and inflammatory cytokines/chemokines (Kanmani and Kim, 2019). Poly-IC12U is an altered form of poly IC. It reduces poly IC-associated toxicity and regulates IFN expression by activating the TLR3 receptor (Martins et al., 2015). As a synthetically derived form of poly-IC, Poly-ICLC can induce strong Th1 cytokine responses, including IL-6, IL-12, TNF-α, IFN-*γ*, and type 1 IFNs (Komal et al., 2021). ARNAX is a synthetic DNA–dsRNA hybrid compound and can activate MDA5 (Komal et al., 2021). ARNAX cannot activate the TLR3 pathway.

Different from ARNAX, poly-IC can activate both TLR3 and MDA5 (Shime et al., 2017). In addition, the inflammatory status of macrophages and DCs can also be changed by poly-IC and RGC100 (Longhi et al., 2009; Gupta et al., 2016; Takeda et al., 2018). Therefore, TLR3 agonists, poly-IC and RGC100, ought to be considered as adjuvants for highly virulent PEDV vaccination.

## 5 Conclusion

As an acute and highly contagious enteric disease of swine, highly virulent PEDV causes high piglet mortality and significant economic losses. However, commercialized vaccines can only provide partial cross-protection against novel highly virulent PEDV strains. The development of new vaccines against highly virulent PEDV including mRNA-LNP, subunit, trypsin-independent, nanoparticle-entrapped killed PEDV, and virus-like particle (VLP) vaccines will help protect the swine industry from the serious challenges posed by highly virulent PEDV infection. Natural compounds and chemical and biochemical source-targeted drugs can enhance the effectiveness of drug-based prevention in controlling highly virulent PEDV. As adjuvants, TLR3 agonists can aid vaccines to compensate for IFN secretory deficiencies to protect pigs from highly virulent PEDV infection. Researchers working on the vaccines and drugs mentioned in this review need more time to complete in-depth studies on vaccines and targeted drugs against highly virulent porcine epidemic diarrhea virus. Continued focus on the ongoing research of these vaccines and drugs will provide valuable scientific information for their application in PEDV control and prevention, once sufficient evidence supports effective strategies for managing the disease.

## Glossary

AVP: Antiviral protein
COE: CO-26 K-equivalent
DCs: Dendritic cells
dsRNA: Double-stranded RNA
ECD: Ectodomain
HP: Highly pathogenic
IKK: Nuclear factor-kappab (ikappab) kinase
IRF3/7: IFN-regulatory factor 3/7
ISGs: IFN-stimulated genes
ISRE: IFN-stimulated response element
KAg: Killed vaccine antigens
KPNA2: Karyopherin α 2
LNP: Lipid nanoparticle
MCs: Mast cells
MDA-5: Melanoma differentiation-associated gene 5
MHC: Major histocompatibility complex
mRNA-LNP: LNP-encapsulated mRNA
N protein: PEDV nucleocapsid (N) protein
NF-κB: Nuclear transcription factor-κB
NPs: Nanoparticles
NTD: N-terminal domain
NTPase: Nucleoside triphosphate hydrolase
ORFs: Open reading frames
PAMPs: Pathogen-associated molecular patterns
PED: Porcine epidemic diarrhea
PEDV: Porcine epidemic diarrhea virus
PEDV-S mRNA-LNP vaccine: mRNA-LNP vaccines encoding a PEDV multiepitope Sm protein
PI3K: Phosphatidylinositol 3-kinase
PKB/AKT: PI3K /protein kinase B
PLGA: Poly (D, L -lactide-co-glycolide)
PLGA-KAg: PLGA nanoparticle–entrapped PEDV KAG
PLP2: Papain-like protease 2
PolyI:C: Polycytidylic acid
PRRs: Pattern-recognition receptors
RdRp: RNA-dependent RNA polymerase
RIG-I: Retinoic acid-inducible gene 1
STAT3: Signal transducer and activator of transcription 3
Sm: Chimeric spike protein
STAT: Signal transducer and activator of transcription
TIR: Toll/IL-1 receptor
TLRs: Toll-like receptors
TLR3: Toll-like receptor 3
TRAF3: Tumor necrosis factor (TNF) receptor-associated factor 3
TRIF: TIR domain-containing adapter-inducing interferon-β;
VLPs: Virus-like particles

## References

[ref1] Abe Y. Fujii K. Nagata N. Takeuchi O. Akira S. Oshiumi H.. (2012). The toll-like receptor 3-mediated antiviral response is important for protection against poliovirus infection in poliovirus receptor transgenic mice. J. Virol. 86, 185–194. doi: 10.1128/jvi.05245-11, PMID: 22072781 PMC3255933

[ref2] Agier J. Żelechowska P. Kozłowska E. Brzezińska-Błaszczyk E. (2016). Expression of surface and intracellular toll-like receptors by mature mast cells. Cent Eur J Immunol 4, 333–338. doi: 10.5114/ceji.2016.65131, PMID: 28450795 PMC5382879

[ref3] Behzadi A. Imani S. Deravi N. Mohammad Taheri Z. Mohammadian F. Moraveji Z.. (2023). Antiviral potential of *Melissa officinalis* L.: a literature review. Nutr. Metab. Insights 16:11786388221146683. doi: 10.1177/11786388221146683, PMID: 36655201 PMC9841880

[ref4] Binjawadagi B. Dwivedi V. Manickam C. Ouyang K. Torrelles J. B. Renukaradhya G. J. (2014a). An innovative approach to induce cross-protective immunity against porcine reproductive and respiratory syndrome virus in the lungs of pigs through adjuvanted nanotechnology-based vaccination. Int. J. Nanomedicine 9, 1519–1535. doi: 10.2147/ijn.S59924, PMID: 24711701 PMC3969340

[ref5] Binjawadagi B. Dwivedi V. Manickam C. Ouyang K. Wu Y. Lee L. J.. (2014b). Adjuvanted poly(lactic-co-glycolic) acid nanoparticle-entrapped inactivated porcine reproductive and respiratory syndrome virus vaccine elicits cross-protective immune response in pigs. Int. J. Nanomedicine 9, 679–694. doi: 10.2147/ijn.S56127 24493925 PMC3908835

[ref6] Bugge M. Bergstrom B. Eide O. K. Solli H. Kjønstad I. F. Stenvik J.. (2017). Surface toll-like receptor 3 expression in metastatic intestinal epithelial cells induces inflammatory cytokine production and promotes invasiveness. J. Biol. Chem. 292, 15408–15425. doi: 10.1074/jbc.M117.784090, PMID: 28717003 PMC5602399

[ref7] Cao L. Ge X. Gao Y. Herrler G. Ren Y. Ren X.. (2015a). Porcine epidemic diarrhea virus inhibits dsRNA-induced interferon-β production in porcine intestinal epithelial cells by blockade of the RIG-I-mediated pathway. Virol. J. 12:127. doi: 10.1186/s12985-015-0345-x, PMID: 26283628 PMC4539884

[ref8] Cao L. Ge X. Gao Y. Ren Y. Ren X. Li G. (2015b). Porcine epidemic diarrhea virus infection induces NF-κB activation through the TLR2, TLR3 and TLR9 pathways in porcine intestinal epithelial cells. J. Gen. Virol. 96, 1757–1767. doi: 10.1099/vir.0.000133, PMID: 25814121

[ref9] Chattopadhyay S. Sen G. C. (2014). dsRNA-activation of TLR3 and RLR signaling: gene induction-dependent and independent effects. J. Interf. Cytokine Res. 34, 427–436. doi: 10.1089/jir.2014.0034, PMID: 24905199 PMC4046345

[ref10] Crawford K. Lager K. M. Kulshreshtha V. Miller L. C. Faaberg K. S. (2016). Status of vaccines for porcine epidemic diarrhea virus in the United States and Canada. Virus Res. 226, 108–116. doi: 10.1016/j.virusres.2016.08.005, PMID: 27545066

[ref11] Dastjerdi A. Carr J. Ellis R. J. Steinbach F. Williamson S. (2015). Porcine epidemic diarrhea virus among farmed pigs, Ukraine. Emerg. Infect. Dis. 21, 2235–2237. doi: 10.3201/eid2112.150272, PMID: 26584081 PMC4672447

[ref12] Deng X. van Geelen A. Buckley A. C. O'Brien A. Pillatzki A. Lager K. M.. (2019). Coronavirus endoribonuclease activity in porcine epidemic diarrhea virus suppresses type I and type III interferon responses. J. Virol. 93:e02000–18. doi: 10.1128/jvi.02000-18, PMID: 30728254 PMC6450110

[ref13] Dortmans J. Li W. van der Wolf P. J. Buter G. J. Franssen P. J. M. van Schaik G.. (2018). Porcine epidemic diarrhea virus (PEDV) introduction into a naive Dutch pig population in 2014. Vet. Microbiol. 221, 13–18. doi: 10.1016/j.vetmic.2018.05.014 29981699 PMC7117506

[ref14] Du L. Tai W. Zhou Y. Jiang S. (2016). Vaccines for the prevention against the threat of MERS-CoV. Expert Rev. Vaccines 15, 1123–1134. doi: 10.1586/14760584.2016.1167603, PMID: 26985862 PMC5097835

[ref15] Dudziak D. Kamphorst A. O. Heidkamp G. F. Buchholz V. R. Trumpfheller C. Yamazaki S.. (2007). Differential antigen processing by dendritic cell subsets in vivo. Science 315, 107–111. doi: 10.1126/science.1136080, PMID: 17204652

[ref16] Fang F. Ooka K. Sun X. Shah R. Bhattacharyya S. Wei J.. (2013). A synthetic TLR3 ligand mitigates profibrotic fibroblast responses by inducing autocrine IFN signaling. J. Immunol. 191, 2956–2966. doi: 10.4049/jimmunol.1300376, PMID: 23956427 PMC3924580

[ref17] Gao Q. Weng Z. Feng Y. Gong T. Zheng X. Zhang G.. (2023). KPNA2 suppresses porcine epidemic diarrhea virus replication by targeting and degrading virus envelope protein through selective autophagy. J. Virol. 97:e0011523. doi: 10.1128/jvi.00115-23, PMID: 38038431 PMC10734479

[ref18] Gong M. Xia X. Chen D. Ren Y. Liu Y. Xiang H.. (2023). Antiviral activity of chrysin and naringenin against porcine epidemic diarrhea virus infection. Front Vet Sci 10:1278997. doi: 10.3389/fvets.2023.1278997, PMID: 38130439 PMC10733469

[ref19] Grasland B. Bigault L. Bernard C. Quenault H. Toulouse O. Fablet C.. (2015). Complete genome sequence of a porcine epidemic diarrhea s gene indel strain isolated in France in december 2014. Genome Announc. 3:e00535–15. doi: 10.1128/genomeA.00535-15, PMID: 26044419 PMC4457056

[ref20] Guo L. Luo X. Li R. Xu Y. Zhang J. Ge J.. (2016). Porcine epidemic diarrhea virus infection inhibits interferon signaling by targeted degradation of STAT1. J. Virol. 90, 8281–8292. doi: 10.1128/jvi.01091-16, PMID: 27384656 PMC5008104

[ref21] Guo W. Wang C. Song X. Xu H. Zhao S. Gu J.. (2024). Immunogenicity and protective efficacy of a trimeric full-length S protein subunit vaccine for porcine epidemic diarrhea virus. Vaccine 42, 828–839. doi: 10.1016/j.vaccine.2024.01.020, PMID: 38220489

[ref22] Gupta S. K. Yadav P. K. Tiwari A. K. Gandham R. K. Sahoo A. P. (2016). Poly (I:C) enhances the anti-tumor activity of canine parvovirus NS1 protein by inducing a potent anti-tumor immune response. Tumour Biol. 37, 12089–12102. doi: 10.1007/s13277-016-5093-z, PMID: 27209409

[ref23] Hsu C. W. Chang M. H. Chang H. W. Wu T. Y. Chang Y. C. (2020). Parenterally administered porcine epidemic diarrhea virus-like particle-based vaccine formulated with CCL25/28 chemokines induces systemic and mucosal immune Protectivity in pigs. Viruses 12:1122. doi: 10.3390/v12101122, PMID: 33023277 PMC7600258

[ref24] Hsueh F. C. Chang Y. C. Kao C. F. Hsu C. W. Chang H. W. (2020). Intramuscular immunization with chemokine-Adjuvanted inactive porcine epidemic diarrhea virus induces substantial protection in pigs. Vaccines (Basel) 8:102. doi: 10.3390/vaccines8010102, PMID: 32102459 PMC7157555

[ref25] Jang G. Lee D. Shin S. Lim J. Won H. Eo Y.. (2023). Porcine epidemic diarrhea virus: an update overview of virus epidemiology, vaccines, and control strategies in South Korea. J. Vet. Sci. 24:e58. doi: 10.4142/jvs.23090, PMID: 37532301 PMC10404706

[ref26] Kanmani P. Kim H. (2019). Immunobiotic strains modulate toll-like receptor 3 agonist induced innate antiviral immune response in human intestinal epithelial cells by modulating IFN regulatory factor 3 and NF-κB signaling. Front. Immunol. 10:1536. doi: 10.3389/fimmu.2019.01536, PMID: 31333667 PMC6618302

[ref27] Karte C. Platje N. Bullermann J. Beer M. Höper D. Blome S. (2020). Re-emergence of porcine epidemic diarrhea virus in a piglet-producing farm in northwestern Germany in 2019. BMC Vet. Res. 16:329. doi: 10.1186/s12917-020-02548-4, PMID: 32912228 PMC7481547

[ref28] Kirchdoerfer R. N. Bhandari M. Martini O. Sewall L. M. Bangaru S. Yoon K. J.. (2021). Structure and immune recognition of the porcine epidemic diarrhea virus spike protein. Structure 29, 385–392.e5. doi: 10.1016/j.str.2020.12.003, PMID: 33378641 PMC7962898

[ref29] Komal A. Noreen M. El-Kott A. F. (2021). TLR3 agonists: RGC100, ARNAX, and poly-IC: a comparative review. Immunol. Res. 69, 312–322. doi: 10.1007/s12026-021-09203-6, PMID: 34145551 PMC8213534

[ref30] Lee D. Kim S. Gim Y. Lee C. (2024). Genotypic characterization of novel S-DEL variants of porcine epidemic diarrhea virus identified in South Korea. Arch. Virol. 169:158. doi: 10.1007/s00705-024-06088-2, PMID: 38970647

[ref31] Leidenberger S. Schröder C. Zani L. Auste A. Pinette M. Ambagala A.. (2017). Virulence of current German PEDV strains in suckling pigs and investigation of protective effects of maternally derived antibodies. Sci. Rep. 7:10825. doi: 10.1038/s41598-017-11160-w, PMID: 28883628 PMC5589859

[ref32] Li B. Du L. Yu Z. Sun B. Xu X. Fan B.. (2017). Poly (d,l-lactide-co-glycolide) nanoparticle-entrapped vaccine induces a protective immune response against porcine epidemic diarrhea virus infection in piglets. Vaccine 35, 7010–7017. doi: 10.1016/j.vaccine.2017.10.054, PMID: 29102169

[ref33] Li L. Li H. Qiu Y. Li J. Zhou Y. Lv M.. (2024). PA-824 inhibits porcine epidemic diarrhea virus infection in vivo and in vitro by inhibiting p53 activation. J. Virol. 98:e0041323. doi: 10.1128/jvi.00413-23, PMID: 38864728 PMC11265451

[ref34] Li Z. Ma Z. Li Y. Gao S. Xiao S. (2020). Porcine epidemic diarrhea virus: molecular mechanisms of attenuation and vaccines. Microb. Pathog. 149:104553. doi: 10.1016/j.micpath.2020.104553, PMID: 33011361 PMC7527827

[ref35] Li Q. Peng O. Wu T. Xu Z. Huang L. Zhang Y.. (2018). PED subunit vaccine based on COE domain replacement of flagellin domain D3 improved specific humoral and mucosal immunity in mice. Vaccine 36, 1381–1388. doi: 10.1016/j.vaccine.2018.01.086, PMID: 29426660

[ref36] Li S. Yang J. Zhu Z. Zheng H. (2020). Porcine epidemic diarrhea virus and the host innate immune response. Pathogens 9:367. doi: 10.3390/pathogens9050367, PMID: 32403318 PMC7281546

[ref37] Li M. Zhang Y. Fang Y. Xiao S. Fang P. Fang L. (2023). Construction and immunogenicity of a trypsin-independent porcine epidemic diarrhea virus variant. Front. Immunol. 14:1165606. doi: 10.3389/fimmu.2023.1165606, PMID: 37033982 PMC10080105

[ref38] Li Y. Zhu Y. Wang Y. Feng Y. Li D. Li S.. (2023). Characterization of RNA G-quadruplexes in porcine epidemic diarrhea virus genome and the antiviral activity of G-quadruplex ligands. Int. J. Biol. Macromol. 231:123282. doi: 10.1016/j.ijbiomac.2023.123282, PMID: 36657537

[ref39] Liang J. Xu W. Gou F. Qin L. Yang H. Xiao J.. (2024). Antiviral activity of flavonol against porcine epidemic diarrhea virus. Virology 597:110128. doi: 10.1016/j.virol.2024.110128, PMID: 38861876

[ref40] Lin H. Chen L. Gao L. Yuan X. Ma Z. Fan H. (2016). Epidemic strain YC2014 of porcine epidemic diarrhea virus could provide piglets against homologous challenge. Virol. J. 13:68. doi: 10.1186/s12985-016-0529-z, PMID: 27103490 PMC4840883

[ref41] Longhi M. P. Trumpfheller C. Idoyaga J. Caskey M. Matos I. Kluger C.. (2009). Dendritic cells require a systemic type I interferon response to mature and induce CD4+ Th1 immunity with poly IC as adjuvant. J. Exp. Med. 206, 1589–1602. doi: 10.1084/jem.20090247, PMID: 19564349 PMC2715098

[ref42] Lu Y. Clark-Deener S. Gillam F. Heffron C. L. Tian D. Sooryanarain H.. (2020). Virus-like particle vaccine with B-cell epitope from porcine epidemic diarrhea virus (PEDV) incorporated into hepatitis B virus core capsid provides clinical alleviation against PEDV in neonatal piglets through lactogenic immunity. Vaccine 38, 5212–5218. doi: 10.1016/j.vaccine.2020.06.009, PMID: 32565343

[ref43] Makadiya N. Brownlie R. van den Hurk J. Berube N. Allan B. Gerdts V.. (2016). S1 domain of the porcine epidemic diarrhea virus spike protein as a vaccine antigen. Virol. J. 13:57. doi: 10.1186/s12985-016-0512-8, PMID: 27036203 PMC4818391

[ref44] Manolova V. Flace A. Bauer M. Schwarz K. Saudan P. Bachmann M. F. (2008). Nanoparticles target distinct dendritic cell populations according to their size. Eur. J. Immunol. 38, 1404–1413. doi: 10.1002/eji.200737984, PMID: 18389478

[ref45] Martins K. A. Bavari S. Salazar A. M. (2015). Vaccine adjuvant uses of poly-IC and derivatives. Expert Rev. Vaccines 14, 447–459. doi: 10.1586/14760584.2015.966085, PMID: 25308798

[ref46] Matsumoto M. Funami K. Tanabe M. Oshiumi H. Shingai M. Seto Y.. (2003). Subcellular localization of toll-like receptor 3 in human dendritic cells. J. Immunol. 171, 3154–3162. doi: 10.4049/jimmunol.171.6.3154, PMID: 12960343

[ref47] Matsumoto M. Oshiumi H. Seya T. (2011). Antiviral responses induced by the TLR3 pathway. Rev. Med. Virol. 21, 67–77. doi: 10.1002/rmv.680, PMID: 21312311

[ref48] Mesonero-Escuredo S. Strutzberg-Minder K. Casanovas C. Segalés J. (2018). Viral and bacterial investigations on the aetiology of recurrent pig neonatal diarrhoea cases in Spain. Porcine Health Manag 4:5. doi: 10.1186/s40813-018-0083-8, PMID: 29632701 PMC5885353

[ref49] Mesquita J. R. Hakze-van der Honing R. Almeida A. Lourenço M. van der Poel W. H. Nascimento M. S. (2015). Outbreak of porcine epidemic diarrhea virus in Portugal, 2015. Transbound. Emerg. Dis. 62, 586–588. doi: 10.1111/tbed.12409, PMID: 26344708 PMC7169791

[ref50] Mohsen M. O. Gomes A. C. Cabral-Miranda G. Krueger C. C. Leoratti F. M. Stein J. V.. (2017). Delivering adjuvants and antigens in separate nanoparticles eliminates the need of physical linkage for effective vaccination. J. Control. Release 251, 92–100. doi: 10.1016/j.jconrel.2017.02.031, PMID: 28257987

[ref51] Monteagudo L. V. Benito A. A. Lázaro-Gaspar S. Arnal J. L. Martin-Jurado D. Menjon R.. (2022). Occurrence of rotavirus a genotypes and other enteric pathogens in diarrheic suckling piglets from Spanish swine farms. Animals (Basel) 12:251. doi: 10.3390/ani12030251, PMID: 35158575 PMC8833434

[ref52] Naumann K. Wehner R. Schwarze A. Petzold C. Schmitz M. Rohayem J. (2013). Activation of dendritic cells by the novel toll-like receptor 3 agonist RGC100. Clin. Dev. Immunol. 2013:283649. doi: 10.1155/2013/283649, PMID: 24454470 PMC3878805

[ref53] Negishi H. Osawa T. Ogami K. Ouyang X. Sakaguchi S. Koshiba R.. (2008). A critical link between toll-like receptor 3 and type II interferon signaling pathways in antiviral innate immunity. Proc. Natl. Acad. Sci. USA 105, 20446–20451. doi: 10.1073/pnas.0810372105, PMID: 19074283 PMC2629334

[ref54] Nguyen N. H. Huynh T. M. Nguyen H. D. Lai D. C. Nguyen M. N. (2023). Epidemiological and genetic characterization of porcine epidemic diarrhea virus in the Mekong Delta, Vietnam, from 2015 to 2017. Arch. Virol. 168:152. doi: 10.1007/s00705-023-05779-6, PMID: 37140665

[ref55] Niu X. Wang Q. (2022). Prevention and control of porcine epidemic diarrhea: the development of recombination-resistant live attenuated vaccines. Viruses 14:1317. doi: 10.3390/v14061317, PMID: 35746788 PMC9227446

[ref56] Park S. J. Moon H. J. Yang J. S. Lee C. S. Song D. S. Kang B. K.. (2007). Sequence analysis of the partial spike glycoprotein gene of porcine epidemic diarrhea viruses isolated in Korea. Virus Genes 35, 321–332. doi: 10.1007/s11262-007-0096-x, PMID: 17436070

[ref57] Puranaveja S. Poolperm P. Lertwatcharasarakul P. Kesdaengsakonwut S. Boonsoongnern A. Urairong K.. (2009). Chinese-like strain of porcine epidemic diarrhea virus, Thailand. Emerg. Infect. Dis. 15, 1112–1115. doi: 10.3201/eid1507.081256, PMID: 19624933 PMC2744260

[ref58] Rai R. C. (2020). Host inflammatory responses to intracellular invaders: review study. Life Sci. 240:117084. doi: 10.1016/j.lfs.2019.117084, PMID: 31759040

[ref59] Rao H. Su W. Zhang X. Wang Y. Li T. Li J.. (2023). Hypericum japonicum extract inhibited porcine epidemic diarrhea virus in vitro and in vivo. Front. Pharmacol. 14:1112610. doi: 10.3389/fphar.2023.1112610, PMID: 37138845 PMC10149974

[ref60] Russo M. Moccia S. Spagnuolo C. Tedesco I. Russo G. L. (2020). Roles of flavonoids against coronavirus infection. Chem. Biol. Interact. 328:109211. doi: 10.1016/j.cbi.2020.109211, PMID: 32735799 PMC7385538

[ref61] Shi P. Su Y. Li R. Liang Z. Dong S. Huang J. (2019). PEDV nsp16 negatively regulates innate immunity to promote viral proliferation. Virus Res. 265, 57–66. doi: 10.1016/j.virusres.2019.03.005, PMID: 30849413 PMC7114654

[ref62] Shime H. Maruyama A. Yoshida S. Takeda Y. Matsumoto M. Seya T. (2017). Toll-like receptor 2 ligand and interferon-γ suppress anti-tumor T cell responses by enhancing the immunosuppressive activity of monocytic myeloid-derived suppressor cells. Onco Targets Ther 7:e1373231. doi: 10.1080/2162402x.2017.1373231, PMID: 29296526 PMC5739553

[ref63] Soto J. A. Gálvez N. M. S. Andrade C. A. Pacheco G. A. Bohmwald K. Berrios R. V.. (2020). The role of dendritic cells during infections caused by highly prevalent viruses. Front. Immunol. 11:1513. doi: 10.3389/fimmu.2020.01513, PMID: 32765522 PMC7378533

[ref64] Stadler J. Zoels S. Fux R. Hanke D. Pohlmann A. Blome S.. (2015). Emergence of porcine epidemic diarrhea virus in southern Germany. BMC Vet. Res. 11:142. doi: 10.1186/s12917-015-0454-1, PMID: 26135732 PMC4487554

[ref65] Steinrigl A. Fernández S. R. Stoiber F. Pikalo J. Sattler T. Schmoll F. (2015). First detection, clinical presentation and phylogenetic characterization of porcine epidemic diarrhea virus in Austria. BMC Vet. Res. 11:310. doi: 10.1186/s12917-015-0624-1, PMID: 26714453 PMC4696200

[ref66] Su M. Shi D. Xing X. Qi S. Yang D. Zhang J.. (2021). Coronavirus porcine epidemic diarrhea virus Nucleocapsid protein interacts with p53 to induce cell cycle arrest in S-phase and promotes viral replication. J. Virol. 95:e0018721. doi: 10.1128/jvi.00187-21, PMID: 34037422 PMC8373254

[ref67] Sun P. Wang M. Li J. Qiu Y. Li H. Lv M.. (2022). Inhibitory effect of Buddlejasaponin IVb on porcine epidemic diarrhea virus in vivo and in vitro. Vet. Microbiol. 272:109516. doi: 10.1016/j.vetmic.2022.109516, PMID: 35901581

[ref68] Takeda K. Akira S. (2004). TLR signaling pathways. Semin. Immunol. 16, 3–9. doi: 10.1016/j.smim.2003.10.003, PMID: 14751757

[ref69] Takeda Y. Takaki H. Fukui-Miyazaki A. Yoshida S. Matsumoto M. Seya T. (2018). Vaccine adjuvant ARNAX promotes mucosal IgA production in influenza HA vaccination. Biochem. Biophys. Res. Commun. 506, 1019–1025. doi: 10.1016/j.bbrc.2018.10.166, PMID: 30404733

[ref70] Theuns S. Conceição-Neto N. Christiaens I. Zeller M. Desmarets L. M. Roukaerts I. D.. (2015). Complete genome sequence of a porcine epidemic diarrhea virus from a novel outbreak in Belgium, January 2015. Genome Announc. 3:e00506–15. doi: 10.1128/genomeA.00506-15, PMID: 25999551 PMC4440965

[ref71] Unterholzner L. Keating S. E. Baran M. Horan K. A. Jensen S. B. Sharma S.. (2010). IFI16 is an innate immune sensor for intracellular DNA. Nat. Immunol. 11, 997–1004. doi: 10.1038/ni.1932, PMID: 20890285 PMC3142795

[ref72] Vidal A. Martín-Valls G. E. Tello M. Mateu E. Martín M. Darwich L. (2019). Prevalence of enteric pathogens in diarrheic and non-diarrheic samples from pig farms with neonatal diarrhea in the north east of Spain. Vet. Microbiol. 237:108419. doi: 10.1016/j.vetmic.2019.108419, PMID: 31585655 PMC7117353

[ref73] Wang P. Bai J. Liu X. Wang M. Wang X. Jiang P. (2020). Tomatidine inhibits porcine epidemic diarrhea virus replication by targeting 3CL protease. Vet. Res. 51:136. doi: 10.1186/s13567-020-00865-y, PMID: 33176871 PMC7656508

[ref74] Wang X. Chen J. Shi D. Shi H. Zhang X. Yuan J.. (2016). Immunogenicity and antigenic relationships among spike proteins of porcine epidemic diarrhea virus subtypes G1 and G2. Arch. Virol. 161, 537–547. doi: 10.1007/s00705-015-2694-6, PMID: 26611909 PMC7087089

[ref75] Wang Y. Huang H. Li D. Zhao C. Li S. Qin P.. (2023). Identification of niclosamide as a novel antiviral agent against porcine epidemic diarrhea virus infection by targeting viral internalization. Virol. Sin. 38, 296–308. doi: 10.1016/j.virs.2023.01.008, PMID: 36702255 PMC10176444

[ref76] Wang J. Zeng X. Yin D. Yin L. Shen X. Xu F.. (2023). In silico and in vitro evaluation of antiviral activity of wogonin against main protease of porcine epidemic diarrhea virus. Front. Cell. Infect. Microbiol. 13:1123650. doi: 10.3389/fcimb.2023.1123650, PMID: 37009514 PMC10050881

[ref77] Wang T. Zheng G. Chen Z. Wang Y. Zhao C. Li Y.. (2024). Drug repurposing screens identify tubercidin as a potent antiviral agent against porcine nidovirus infections. Virus Res. 339:199275. doi: 10.1016/j.virusres.2023.199275, PMID: 38008220 PMC10730850

[ref78] Weng L. Weersink A. Poljak Z. de Lange K. von Massow M. (2016). An economic evaluation of intervention strategies for porcine epidemic diarrhea (PED). Prev. Vet. Med. 134, 58–68. doi: 10.1016/j.prevetmed.2016.09.018, PMID: 27836046

[ref79] Witczak P. Brzezińska-Błaszczyk E. Agier J. (2020). The response of tissue mast cells to TLR3 ligand poly(I:C) treatment. J Immunol Res 2020, 1–13. doi: 10.1155/2020/2140694, PMID: 32185237 PMC7060451

[ref80] Wu M. Zhang Q. Yi D. Wu T. Chen H. Guo S.. (2020). Quantitative proteomic analysis reveals antiviral and anti-inflammatory effects of Puerarin in piglets infected with porcine epidemic diarrhea virus. Front. Immunol. 11:169. doi: 10.3389/fimmu.2020.00169, PMID: 32174911 PMC7055472

[ref81] Xu X. Du L. Fan B. Sun B. Zhou J. Guo R.. (2020). A flagellin-adjuvanted inactivated porcine epidemic diarrhea virus (PEDV) vaccine provides enhanced immune protection against PEDV challenge in piglets. Arch. Virol. 165, 1299–1309. doi: 10.1007/s00705-020-04567-w, PMID: 32253616 PMC7223252

[ref82] Xu X. Gao S. Zuo Q. Gong J. Song X. Liu Y.. (2024). Enhanced in vitro antiviral activity of Ivermectin-loaded nanostructured lipid carriers against porcine epidemic diarrhea virus via improved intracellular delivery. Pharmaceutics 16:601. doi: 10.3390/pharmaceutics16050601, PMID: 38794264 PMC11125651

[ref83] Xu Z. Liu Y. Peng P. Liu Y. Huang M. Ma Y.. (2020). Aloe extract inhibits porcine epidemic diarrhea virus in vitro and in vivo. Vet. Microbiol. 249:108849. doi: 10.1016/j.vetmic.2020.108849, PMID: 32979750 PMC7491386

[ref84] Yang S. Huang X. Li S. Wang C. Jansen C. A. Savelkoul H. F. J.. (2023). Linoleic acid: a natural feed compound against porcine epidemic diarrhea disease. J. Virol. 97:e0170023. doi: 10.1128/jvi.01700-23, PMID: 38009930 PMC10734519

[ref85] Yang Q. Shu H. B. (2020). Deciphering the pathways to antiviral innate immunity and inflammation. Adv. Immunol. 145, 1–36. doi: 10.1016/bs.ai.2019.11.001, PMID: 32081195

[ref86] Zhao Y. Fan B. Song X. Gao J. Guo R. Yi C.. (2024). PEDV-spike-protein-expressing mRNA vaccine protects piglets against PEDV challenge. MBio 15:e0295823. doi: 10.1128/mbio.02958-23, PMID: 38231557 PMC10865985

[ref87] Zhuang L. Zhao Y. Shen J. Sun L. Hao P. Yang J.. (2025). Advances in porcine epidemic diarrhea virus research: genome, epidemiology, vaccines, and detection methods. Discov Nano 20:48. doi: 10.1186/s11671-025-04220-y, PMID: 40029472 PMC11876513

